# Convergence analysis of efficient online learning in Bayesian spiking neurons

**DOI:** 10.1186/1471-2202-13-S1-P129

**Published:** 2012-07-16

**Authors:** Andre Van Schaik, Levin Kuhlmann, Michael Hauser-Raspe, Jonathan Manton, Jonathan Tapson, David B Grayden

**Affiliations:** 1 MARCS Institute, University of Western Sydney, Penrith, Australia; 2 NeuroEngineering Laboratory, Department of Electrical and Electronic Engineering, The University of Melbourne, 3010, Victoria, Australia

## 

Bayesian spiking neurons (BSNs) provide a probablisitic and intuitive interpretation of how spiking neurons could work and have been shown to be equivalent to leaky integrate-and-fire neurons under certain conditions [1]. The study of BSNs has been restricted mainly to small networks because online learning, which currently involves a maximum-likelihood-expectation-maximisation (ML-EM) approach [2,3], is quite slow. Here a new approach to estimating the parameters of Bayesian spiking neurons, referred to as fast learning (FL), is presented and compared to online ML-EM learning.

Learning in a BSN is local to the neuron and involves estimation of the transition rate and observation rate parameters of an underlying implicit hidden Markov model (HMM), the hidden state of which the BSN output encodes [1]. Rather than estimating the parameters by maximizing the log-likelihood of the hidden states and the synaptic observations given the parameters as is done in ML-EM [2,3], the FL algorithm directly calculates statistics upon which the parameters depend. This is achieved by taking advantage of the relationship between the log-odds ratio of the hidden state computed by the BSN and the probability that the hidden state is ‘on’ given the past synaptic observations, .

Online learning in a two BSN neuron hierarchy is explored, where the first neuron receives N=20 synapses driven by Poisson processes and the second neuron receives input from only the first neuron. Simulations were performed for a fixed set of transition rates and observation rates for 10 different perturbations of the initial transition and observation rate estimates: ±0-20%, ±20-40%,..., ±180-200%. Initial rates were not allowed to go below 10^-6^. Each perturbation condition was simulated 100 times by randomly selecting the initial parameter values.

Although the FL algorithm is not as exact as the ML-EM at estimating the true parameter values for small perturbations of the initial rate estimates relative to the true rates, the FL algorithm is able to reliably estimate the parameters for initial perturbations of up to 200%, whereas the ML-EM algorithm estimates begin to deviate after perturbations of about 40-60%. Moreover, the simplicity of the FL algorithm means that it runs on the order of 25 times faster than the ML-EM implementation considered. These results hold true for both the first and second neurons in the two BSN neuron hierarchy. For the first neuron in the hierarchy, the RMS difference in the time series of the probability  calculated for the estimated and the true parameter values, follows a similar pattern to the parameter estimates when the FL and ML-EM algorithms are compared, with average RMS errors of 0.2% obtained for the FL algorithm across the range of perturbations studied.

Although we do not have a formal proof of convergence of the FL algorithm, we conclude that the FL algorithm can stably estimate the parameters over a large range of initial perturbations and it can do this very quickly. Thus the FL algorithm makes online learning in networks of BSNs much more tractable.
